# Clinical analysis of 48 patients with carotid web diagnosed by digital subtraction angiography

**DOI:** 10.3389/fneur.2026.1862595

**Published:** 2026-07-30

**Authors:** Jing Huang, Zhejing Ding, Guomin Huang, Qiang Zhou, Guangsen Cheng, Yu Liu, Xiong Zhang, Yu-sheng Zhang

**Affiliations:** 1 Department of Cerebrovascular Diseases, Zhuhai Clinical Medical College of Jinan University (Zhuhai People’s Hospital, The Affiliated Hospital of Beijing Institute of Technology, The First Affiliated Hospital of the Faculty of Medicine Macau University of Science and Technology), Zhuhai, Guangdong, China; 2 Laboratory of Drug Discovery from Natural Resources and Industrialization, School of Pharmacy, Faculty of Medicine, Macau University of Science and Technology, Taipa, Macau SAR, China; 3 Department of Neurology, Maoming People’s Hospital, Maoming, Guangdong, China; 4 Department of Neurology, The Sixth Affiliated Hospital of Jinan University, Dongguan Eastern Central Hospital, Dongguan, Guangdong, China; 5 Department of Neurology and Stroke Center, The First Affiliated Hospital of Jinan University, Guangzhou, Guangdong, China

**Keywords:** Bradford-Hill criteria, carotid web, cryptogenic stroke, digital subtraction angiography, ischemic stroke, multidisciplinary team, subweb thrombus

## Abstract

**Objective:**

To investigate the clinical characteristics, imaging features, relationship with ischemic stroke, treatment strategies, and follow-up outcomes of patients with carotid web (CaW) diagnosed by digital subtraction angiography (DSA), in order to improve the understanding and clinical management of CaW.

**Methods:**

A retrospective analysis was conducted on the clinical data of 48 patients diagnosed with CaW by DSA in the Department of Cerebrovascular Diseases, Zhuhai People’s Hospital from December 2021 to June 2025. Data included baseline characteristics, imaging findings, treatment methods, and follow-up results. All clinical outcomes (ischemic stroke, transient ischemic attack, recurrent ipsilateral ischemic event) were defined *a priori* according to the American Heart Association/American Stroke Association 2013 (stroke) and 2009 (tissue-based TIA) criteria, and follow-up imaging (carotid Doppler at 6 and 12 months, with brain MRI ± MRA/CTA when clinically indicated) was standardized across all patients.

**Results:**

The 48 patients aged from 28 to 83 years, with a mean age of (60.81 ± 11.58). There were 26 males (54.17%) and 22 females (45.83%). All lesions were located at the carotid bifurcation or the origin of the internal carotid artery. The typical DSA manifestation was a thin, linear or membranous filling defect originating from the vessel wall. Among the 48 patients, 13 had a history of smoking, 12 of alcohol consumption, 17 had hypertension, 18 had hyperlipidemia, 7 had diabetes mellitus, 2 had hyperhomocysteinemia, and 4 had hyperuricemia. There were 24 cases of left-sided CaW, 21 cases of right-sided CaW, and 3 cases of bilateral CaW. Subweb thrombus was present in 22 cases (45.83%). Acute ischemic stroke events occurred in 9 cases (18.75%). Treatment modalities included antiplatelet or anticoagulant therapy alone, endovascular interventional therapy, and surgical resection. Specifically, 34 patients received medication alone, 1 underwent emergency endovascular thrombectomy, 1 underwent carotid endarterectomy, 4 underwent carotid artery stenting, and 8 received no treatment. Patients were followed up for 3 to 12 months. One patient in the medication group experienced recurrence of ipsilateral cerebral infarction, while no ischemic events occurred in the remaining patients. “Recurrence” was defined as a new ipsilateral ischemic event occurring ≥24 h after the index event and meeting the AHA/ASA 2013 criteria. All patients completed the 12 month clinical follow-up; loss-to-follow-up was 0%.

**Conclusion:**

In this retrospective, non-randomized cohort, the observed rate of recurrent ipsilateral ischemic events was numerically lower among patients who received active endovascular treatment (0/6) than among those who received medical therapy alone (1/34). However, given the very small number of patients who received active intervention, the short follow-up (3–12 months), and the non-randomized, indication-based treatment allocation, no causal inference can be drawn from these data regarding the efficacy of endovascular treatment in preventing stroke recurrence. The observed difference should be interpreted as hypothesis-generating only and requires confirmation in adequately powered prospective randomized controlled trials. Carotid web is an important risk factor for cryptogenic stroke across all age groups. DSA remains the diagnostic gold standard. For patients who have already experienced ischemic events or who have subweb thrombus, active endovascular treatment represents a potentially useful therapeutic option that warrants further evaluation.

## 1 Introduction

Carotid web (CaW) is a thin, membranous morphological structure located on the posterolateral wall of the carotid bulb, with its medial edge free, presenting as fan-shaped or ribbon-like in morphology. It is currently widely regarded as a rare anatomical variant of fibromuscular dysplasia (FMD) (1, 2). In recent years, CaW has gained increasing attention from neurologists and interventional physicians as a non-negligible causative factor for cryptogenic stroke, particularly as a potential etiology in young stroke patients (3), including cases initially classified as “cryptogenic” but later attributed to carotid bulb webs (2). Its pathogenic mechanism is likely related to the morphological structure of the web, which predisposes to local hemodynamic abnormalities, blood stasis, and vortex formation, thereby facilitating secondary thrombus formation. Emboli from such thrombi can ultimately lead to arterio-arterial embolism events (1). Despite the substantial global burden of stroke (4), conventional examinations can easily miss the diagnosis, digital subtraction angiography (DSA), with its high spatial and temporal resolution, can clearly display this subtle structure and the hemodynamic changes it induces, making it the “gold standard” for diagnosing CaW.

Despite this growing recognition, several important gaps in the current understanding of CaW persist. First, most published cohorts are derived from Western populations, particularly from France, the United States, and Canada, while data from Asian populations—including Chinese patients—remain limited to small case series (5, 6). Ethnic and geographic differences in CaW prevalence, morphology, and stroke risk have been hypothesized but remain poorly characterized (5, 7). Second, the natural history of CaW, in particular the annualized risk of ipsilateral stroke in untreated asymptomatic lesions, is still unknown; reported recurrence rates range widely (0–30%), reflecting heterogeneity in study design, follow-up duration, and population selection (6, 8). Third, the diagnostic criteria for CaW are not standardized, and inter-observer agreement has rarely been quantified (6, 9). Fourth, the optimal treatment strategy remains undefined: existing guidelines provide no specific recommendations for CaW, and there are there are no randomized controlled trials comparing medical therapy, endovascular intervention, and surgical resection (10), endovascular intervention, and surgical resection (11, 12). In particular, it remains uncertain whether active intervention is superior to medical therapy alone for symptomatic CaW and whether surveillance alone is sufficient for incidentally detected asymptomatic CaW. Finally, the prevalence of subweb thrombus—increasingly recognized as the central mechanism linking CaW to embolic stroke—and its prognostic implications have been reported in only a few small imaging series (6). Collectively, these gaps justify additional well-characterized, DSA-based cohorts that integrate standardized diagnostic criteria, structured follow-up, and pre-specified treatment algorithms.

To address these gaps, the present study aimed to: (i) characterize the demographic, clinical, and imaging features of CaW in a relatively large, consecutive, single-center Chinese cohort definitively diagnosed by DSA—the imaging gold standard; (ii) evaluate the prevalence and imaging predictors of subweb thrombus; (iii) describe the treatment strategies actually used and their short-term outcomes under a pre-specified institutional multidisciplinary team (MDT) protocol; and (iv) explore the clinical and imaging features associated with stroke recurrence during follow-up. By integrating standardized diagnostic criteria, structured clinical and imaging follow-up, and a pre-specified MDT-based treatment protocol, this study is intended to provide hypothesis-generating evidence to inform the design of future prospective studies and randomized controlled trials on CaW management.

## 2 Materials and methods

### 2.1 Study population

A retrospective collection and analysis was performed on patients, reported in accordance with the STROBE statement (13) who underwent DSA, reported in accordance with the STROBE statement (14) examination in the Department of Cerebrovascular Diseases, Zhuhai People’s Hospital from December 2021 to June 2025.

Between December 2021 and June 2025, all patients who underwent digital subtraction angiography (DSA) at the Department of Cerebrovascular Diseases, Zhuhai People’s Hospital, were retrospectively screened through the institutional neuro-interventional procedure database and the Hospital Information System (a routinely-collected data source) (15) (a routinely-collected data source) (14) (HIS). Indications for DSA included: (i) suspected carotid web (CaW) identified on prior non-invasive imaging—namely carotid color Doppler ultrasound, computed tomography angiography (CTA), and/or magnetic resonance angiography (MRA); (ii) ischemic stroke or transient ischemic attack (TIA) of undetermined etiology after standard etiological work-up; and (iii) unexplained neurological symptoms (e.g., persistent dizziness or headache) requiring exclusion of cerebrovascular lesions. All DSA examinations were performed using a standardized protocol that included at least two oblique sagittal projections of the carotid bifurcation to optimize visualization of the posterior wall.

To minimize selection bias, only consecutive patients with a confirmed DSA diagnosis of CaW were enrolled. All DSA images were independently reviewed by two senior neuro-interventional physicians (each with more than 10 years of experience in cerebrovascular intervention) who were blinded to the patients’ clinical information. Discrepancies were resolved by the pre-specified 5-step adjudication algorithm described in sub-section 2.7. The final cohort comprised 48 patients.

Inclusion criteria: The diagnosis of CaW was established according to the following pre-specified imaging criteria, adapted from Zhang et al. (6): (i) on axial DSA projections, a thin linear intraluminal filling defect along the posterolateral wall of the carotid bulb or proximal internal carotid artery (ICA); (ii) on oblique sagittal DSA projections, a shelf-like or membranous protrusion extending from the vessel wall into the lumen, with its free edge typically pointing superiorly; (iii) absence of atherosclerotic calcification, irregular plaque, ulceration, or dissection flap; (iv) preserved distal vessel caliber without significant stenosis (NASCET < 50%) (16, 17) [NASCET Steering Committee, Stroke 1991]; and (v) contrast stasis or delayed wash-out in the angle between the web and the posterior wall of the carotid bulb, observed on at least two consecutive frames.

Exclusion criteria were predefined and applied as follows. (1) Diagnostic exclusion criteria: (a) inconclusive or indeterminate CaW diagnosis due to suboptimal image quality or morphology mimicking atherosclerotic plaque, spontaneous carotid dissection, or intraluminal thrombus without a definable web; (b) coexistence of high-grade ipsilateral carotid stenosis (≥50%, NASCET criteria) that precluded clear assessment of the web. (2) Clinical exclusion criteria: (a) prior carotid intervention (carotid artery stenting or carotid endarterectomy) on the affected side; (b) incomplete clinical records or missing baseline imaging preventing comprehensive evaluation. (3) Follow-up exclusion criteria: loss to follow-up within 3 months after DSA.

#### 2.1.1 Categorization of excluded patients

**Figure 1:**
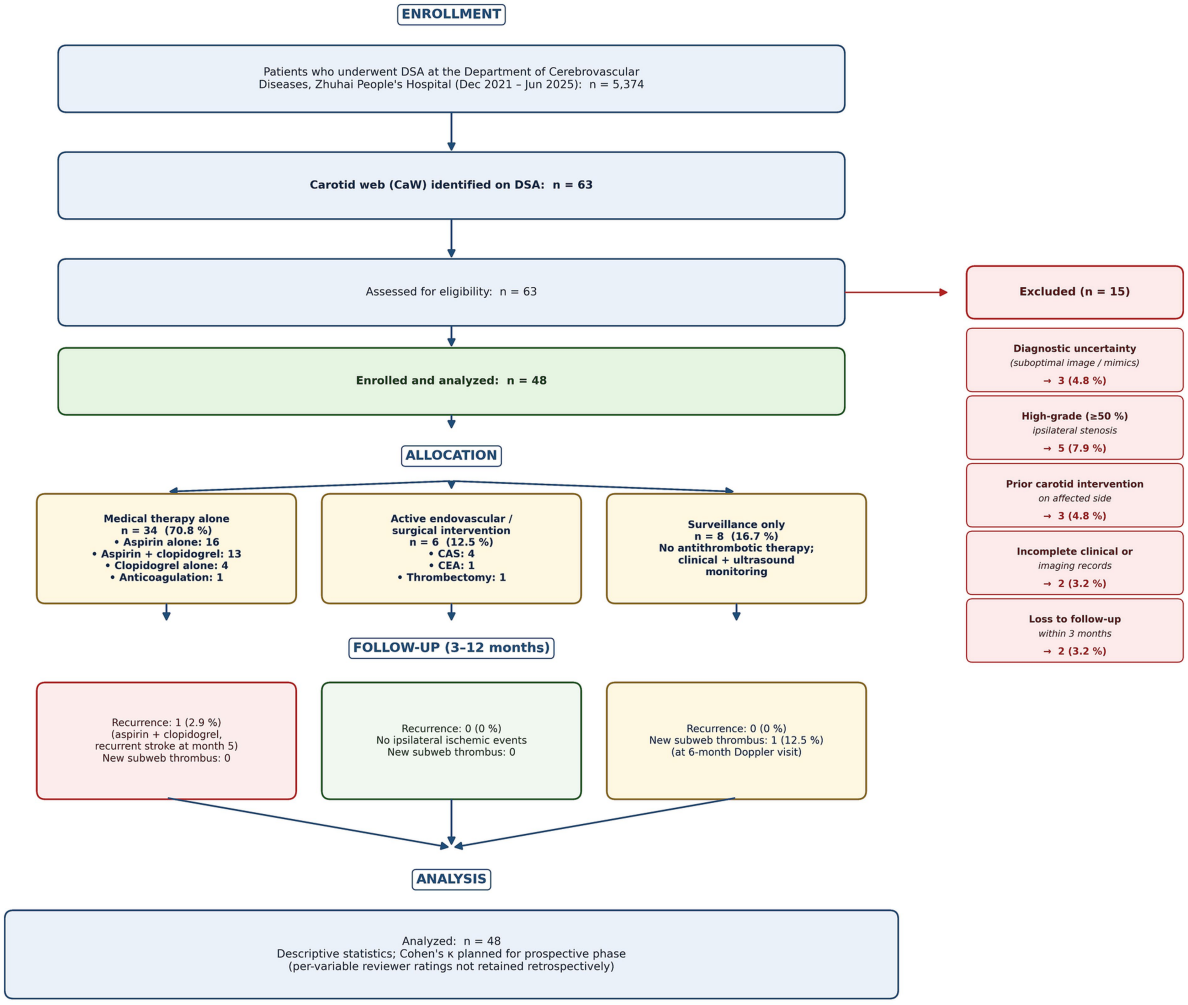
Patient inclusion and exclusion flow diagram. Between December 2021 and June 2025, 5,374 patients underwent DSA at the Department of Cerebrovascular Diseases, Zhuhai People’s Hospital. After excluding 15 patients who did not meet the inclusion criteria or met exclusion criteria (categorized in Table 1), 48 patients with carotid web were included in the final analysis. Of these, 34 received medical therapy alone, 6 received active endovascular or surgical intervention, and 8 were managed with surveillance only. Boxes represent patient cohorts at each stage; arrows indicate flow direction. Percentages for exclusion reasons are computed relative to the 63 patients with confirmed CaW on DSA (15/63 = 23.8%). Percentages for treatment arms are computed relative to the 48 enrolled patients.

A CONSORT-style patient flow diagram summarizing the screening, inclusion, and exclusion process is provided in Figure 1. In brief, of the 5,374 patients who underwent DSA during the study period, 63 were initially diagnosed with CaW; after applying the above inclusion and exclusion criteria, 48 patients were finally included in the analysis. A breakdown of excluded patients by reason is provided in Table 1.

**Table 1:** Number of patients excluded after DSA diagnosis of CaW, by reason.

Reason for exclusion	*n* (% of patients screened with CaW)
Diagnostic uncertainty (suboptimal image quality/mimics)	3 (4.8%)
High-grade (≥50%) ipsilateral carotid stenosis precluding assessment	5 (7.9%)
Prior carotid intervention on the affected side	3 (4.8%)
Incomplete clinical or imaging records	2 (3.2%)
Loss to follow-up within 3 months	2 (3.2%)
Total excluded	15 (23.8% of 63 screened with CaW)

### 2.2 Study methods

Collected patients’ data included baseline information (age, gender, traditional risk factors for cerebrovascular disease), main clinical symptoms, imaging examination results (carotid color Doppler ultrasound, brain MR plain scan, DSA), treatment methods (medication or endovascular intervention/surgery), and outpatient or telephone follow-up results.

#### 2.2.1 DSA acquisition protocol

All DSA examinations were performed on a biplane digital subtraction angiography system, following the STARD 2015 reporting guidelines (18), following the STARD 2015 reporting guidelines (19) (Siemens Artis zee biplane or Philips Allura Xper FD20; Siemens Healthineers/Philips Healthcare). Selective catheterization of the bilateral common carotid arteries (CCA) and internal carotid arteries (ICA) was performed using a 5 Fr diagnostic catheter via a transfemoral or transradial approach. For each carotid bifurcation, the following standardized projections were obtained: (1) anteroposterior (AP) view; (2) true lateral view; (3) ipsilateral oblique view at 30 °–45 °; and (4) contralateral oblique view at 30 °–45 °, with additional magnified and dynamic runs as required. Iohexol (350 mg I/mL) was injected at a rate of 4–6 mL/s for a total of 8–10 mL per run. Image acquisition was performed at 4 frames/s for the first 3 s and 2 frames/s thereafter. In every patient, at least two dedicated oblique sagittal projections of the carotid bulb were acquired to optimize visualization of the posterolateral wall, the most common location of CaW.

Two senior neuro-interventional physicians (each with more than 10 years of experience in cerebrovascular intervention) independently reviewed all DSA images while blinded to the patients’ clinical information, baseline characteristics, and final diagnosis. Discrepancies regarding the presence, location, or morphology of the CaW, as well as the presence of subweb thrombus, were resolved by the 5-step adjudication algorithm described in sub-section 2.7. The number of cases requiring adjudication at each step is reported as a descriptive measure of inter-observer agreement. Formal Cohen’s *κ* coefficients with 95% confidence intervals are planned for the prospective phase of this study, once per-variable reviewer ratings are systematically captured in a structured case-report form.

#### 2.2.2 Criteria for subweb thrombus

Subweb thrombus was diagnosed when both of the following were fulfilled: (a) on carotid color Doppler ultrasound, an echogenic, partially mobile or layered intraluminal mass adherent to the luminal surface of the web, with absent or markedly reduced intra-thrombus Doppler signal; and (b) on DSA, a non-enhancing filling defect located beneath the web and persisting for ≥2 cardiac cycles, after excluding contrast-mixing artifacts. A single positive finding on either modality was considered diagnostic only if corroborated by the other modality. Because Doppler ultrasound is operator-dependent and prone to flow-related artifacts in the low-flow region distal to a CaW, Doppler-based diagnosis of subweb thrombus was considered definitive only when corroborated by a concordant DSA filling defect; otherwise, contrast-enhanced ultrasound (CEUS) was used as an additional confirmatory test (see sub-section 2.6).

#### 2.2.3 Treatment decision-making protocol

Treatment allocation was determined by a fixed institutional multidisciplinary team (MDT) comprising at least two senior neuro-interventional physicians (each with >10 years of experience), a vascular neurologist, and a vascular surgeon. The MDT reviewed all clinical, laboratory, and imaging data before recommending a treatment strategy, and the same protocol was applied throughout the entire study period (December 2021–June 2025) without modification. Treatment was not randomized; however, all decisions were made according to the following pre-specified criteria.

##### 2.2.3.1 Indication for medical therapy alone (antiplatelet or anticoagulant)

Patients were assigned to medical therapy alone if they met at least one of the following: (1) Asymptomatic CaW without subweb thrombus on carotid Doppler ultrasound or DSA; (2) Symptomatic CaW (i.e., CaW ipsilateral to a recent ischemic stroke or TIA) without evidence of recurrent ischemic events after at least 4 weeks of standard antithrombotic therapy; (3) Mild ischemic events (TIA or minor stroke with NIHSS ≤3) without subweb thrombus; (4) High surgical or procedural risk (e.g., severe cardiopulmonary comorbidity, contraindications to dual antiplatelet therapy, or refusal to undergo invasive treatment after informed consent).

##### 2.2.3.2 Indication for active intervention

Active intervention was defined *a priori* as any revascularization procedure specifically targeting the CaW, namely carotid artery stenting (CAS), carotid endarterectomy (CEA), or surgical web resection, as well as any emergent procedure (mechanical thrombectomy) performed to treat a CaW-related acute ischemic stroke. Diagnostic DSA alone was not classified as an active intervention. Active intervention was recommended when at least one of the following criteria was met: (1) Recurrent ipsilateral ischemic stroke or TIA despite ≥ 4 weeks of standard antithrombotic therapy; (2) Documented subweb thrombus on carotid Doppler ultrasound and/or DSA, particularly if mobile, recurrent, or large (≥50% of the web length); (3) Progressive enlargement of the web on serial imaging during follow-up; (4) Coexisting hemodynamically significant ipsilateral carotid stenosis (NASCET ≥50%) attributable to the web or to combined web/atherosclerotic disease; (5) Acute large-vessel occlusion secondary to CaW-related embolism requiring emergency mechanical thrombectomy, with or without subsequent stenting of the culprit lesion.

##### 2.2.3.3 Choice between CAS, CEA, surgical web resection, and mechanical thrombectomy

Once the indication for active intervention was established, the choice between CAS, CEA, surgical web resection, and mechanical thrombectomy was at the discretion of the treating MDT, taking into account lesion morphology, anatomical accessibility, plaque/catheter accessibility, the patient’s comorbidities, and the operator’s expertise. All procedural details were recorded in a prospectively maintained neuro-interventional registry.

##### 2.2.3.4 Standardization

To minimize operator-dependent variability, all treatment decisions were reviewed at the weekly MDT meeting, and the final decision was documented in the electronic medical record using a standardized template that explicitly listed the fulfilled criterion (or criteria) for either medical therapy alone or active intervention.

#### 2.2.4 Outcome definitions

All clinical outcomes were defined *a priori* according to the following standardized criteria. (a) Ischemic stroke was defined according to the American Heart Association/American Stroke Association (AHA/ASA) 2013 expert-consensus definition (20, 21), which was developed in continuity with earlier WHO MONICA criteria (22, 23), as an episode of acute focal neurological dysfunction of presumed vascular origin lasting ≥24 h, or lasting <24 h but associated with new ischemic lesions on brain CT or MRI, and not attributable to another cause. Stroke severity was graded using the National Institutes of Health Stroke Scale (NIHSS) (11, 24) at admission and discharge, and functional outcome using the modified Rankin Scale (mRS) (10, 25) at discharge and at each follow-up visit. (b) Transient ischemic attack (TIA) was defined using the tissue-based definition of the AHA/ASA 2009 scientific statement (26, 27): a brief episode of neurological dysfunction caused by focal brain, spinal cord, or retinal ischemia, with clinical symptoms typically lasting <1 h and without evidence of acute infarction on brain MRI (including diffusion-weighted imaging). All patients with suspected TIA underwent brain MRI with DWI within 7 days of symptom onset. (c) Recurrent ipsilateral ischemic stroke was defined as a new ischemic stroke occurring ≥24 h after the index event (or, if no index event, after the diagnostic DSA), affecting the vascular territory supplied by the carotid artery harboring the CaW, and confirmed by brain MRI with DWI. Recurrent TIA was defined analogously as a new TIA meeting criterion (b) and occurring in the ipsilateral vascular territory. The combined endpoint “recurrent ipsilateral ischemic event” comprised recurrent stroke and recurrent TIA. (d) New subweb thrombus on imaging was defined as a thrombus identified on carotid color Doppler ultrasound and/or DSA meeting the criteria described in sub-section 2.2 that was not present on the baseline imaging study. (e) Hemorrhagic complications were classified according to the Thrombolysis In Myocardial Infarction (TIMI) criteria (28, 29) for intracranial hemorrhage and the Global Utilization of Streptokinase and t-PA for Occluded Arteries (GUSTO) criteria for systemic bleeding. All outcomes were adjudicated by an independent clinical-event committee comprising two vascular neurologists and one neuro-interventional physician who were blinded to treatment allocation.

#### 2.2.5 Follow-up protocol

All patients were enrolled in a standardized, pre-specified follow-up program that was applied uniformly to every patient regardless of treatment allocation. (a) Clinical follow-up: outpatient visit or structured telephone interview at 3, 6, and 12 months after the diagnostic DSA (or after the index intervention, whichever occurred later). At each visit, the following data were systematically collected using a standardized case-report form: any new neurological symptoms (stroke, TIA, amaurosis fugax); adherence to antithrombotic therapy; bleeding events; and mRS score. (b) Imaging follow-up: carotid color Doppler ultrasound was performed at 6 and 12 months after enrollment (or earlier if clinically indicated). Brain MRI with DWI was performed whenever a recurrent neurological event was reported, and MRA or CTA of the cervical carotid arteries was performed in any patient with new ischemic symptoms or new subweb thrombus on Doppler. Repeat DSA was performed only when endovascular re-intervention was considered. (c) Standardization: the same follow-up schedule, imaging modalities, and case-report form were applied to all patients, irrespective of treatment group. Compliance with the schedule and reasons for any deviation were documented. Loss-to-follow-up was defined as failure to complete the 12-month clinical assessment for any reason; patients lost to follow-up were compared with the remaining cohort with respect to baseline characteristics to assess potential attrition bias.

#### 2.2.6 Carotid Doppler ultrasound protocol and differentiation of thrombus from artifact

All carotid color Doppler ultrasound examinations were performed on dedicated vascular ultrasound systems (Philips EPIQ 7 or GE LOGIQ E10) using a high-frequency linear-array transducer (5–12 MHz), with standardized low PRF (≤2,000 Hz), low wall filter (≤100 Hz), and optimized color gain following the SRU Consensus Conference (30) and WFN velocity criteria (31). Spectral Doppler waveforms were obtained with an angle of insonation <60 °. Subweb thrombus was diagnosed only when all four of the following criteria were met: (i) a discrete, layered, partially mobile echogenic mass adherent to the web, with absent intra-thrombus Doppler signal; (ii) concordant DSA filling defect beneath the web; (iii) absence of low-flow artifacts; and (iv) consensus by two independent sonographers. Indeterminate Doppler findings (*n* = 2) were classified as “no thrombus” and underwent CEUS (SonoVue; EFSUMB 2017 guidelines (32)) only if clinically indicated. All studies were independently reviewed by two senior vascular sonographers blinded to clinical information.

#### 2.2.7 Adjudication of disagreements in DSA image review

For each patient, the two reviewing physicians independently completed a structured assessment form following established frameworks for consensus interpretation (33) and key concepts of interrater reliability (34) covering the following pre-specified imaging variables: (i) presence versus absence of CaW; (ii) location (carotid bulb vs. proximal ICA; right, left, or bilateral); (iii) morphological classification (shelf-like vs. membrane-like vs. finger-like); (iv) presence versus absence of subweb thrombus; (v) presence versus absence of coexisting ipsilateral carotid stenosis and its NASCET grade; and (vi) presence versus absence of contrast stasis or delayed wash-out distal to the web.

A “disagreement” was defined as any discrepancy between the two reviewers on any of the six variables listed above. Disagreements were resolved using the following pre-specified, stepwise adjudication algorithm.

Disagreements were resolved using the following pre-specified stepwise algorithm: (1) independent blinded re-review after ≥2 weeks; (2) if persistent, open consensus discussion; (3) if unresolved, arbitration by a third senior neuro-interventional physician (>15 years’ experience) blinded to prior assessments; (4) if disagreement persisted among all three, majority-vote rule applied; cases in which no two of three reviewers agreed on the primary diagnosis (presence vs. absence of CaW) were excluded.

For each of the six imaging variables, the proportion of cases requiring Step 2 or higher, the proportion requiring third-reviewer arbitration (Step 3 or 4), and the number of non-resolvable cases (Step 5) are reported in the Results section.

### 2.3 Statistical analysis

SPSS 26.0 was used. Continuous data are mean ± SD, and count data are expressed as number (percentage). Inter-observer agreement for categorical imaging variables was assessed descriptively, as the proportion of cases requiring adjudication at each step of the algorithm described in sub-section 2.7. Cases flagged as “non-resolvable” at Step 5 are listed individually. Formal Cohen’s *κ* coefficients with 95% confidence intervals are planned for the prospective phase of this study, in which per-variable reviewer ratings will be captured systematically.

## 3 Results

### 3.1 Baseline patient characteristics

The 48 patients aged from 28 to 83 years, with a mean age of (60.81 ± 11.58) years. There were 26 males (54.17%) and 22 females (45.83%). Among the 48 patients, 13 (27.1%) had a history of smoking, 12 (25%) alcohol consumption, 17 (35.4%) had hypertension, 18 (37.5%) had hyperlipidemia, 7 (14.6%) had diabetes mellitus, 2 (4.2%) had hyperhomocysteinemia, and 4 (8.3%) had hyperuricemia. Clinical manifestations were diverse, with dizziness and headache being the most common (16 cases, 33.3%), followed by ischemic events (9 cases, 18.8% of TIA/stroke clearly caused by or highly related to the web). Another 23 cases (47.9%) were discovered incidentally during physical examination or examination for other diseases (Table 2). Of the 23 incidentally detected asymptomatic cases, 8 were managed with surveillance only (no antithrombotic therapy), 14 received antiplatelet therapy (single agent in 11, dual in 3), and 1 received anticoagulation, at the discretion of the treating physician and in accordance with the institutional MDT protocol described in sub-section 2.3.

**Table 2:** Baseline patient characteristics.

Total number	48
Male/Female	26/22
Age, years, median (IQR)	28–83 (60.81 ± 11.58)
Smoking (%)	13 (27.1%)
Alcohol, *n* (%)	12 (25%)
Hypertension, *n* (%)	17 (35.4%)
Hyperlipidemia, *n* (%)	18 (37.5%)
Diabetes, *n* (%)	7 (14.6%)
High homocysteine, *n* (%)	2 (4.2%)
Hyperuricemia, *n* (%)	4 (8.3%)
Reason for visit	
Dizziness/Headache, *n* (%)	16 (33.3%)
Ischemic events, *n* (%)	9 (18.8)
Exam or other check-up, *n* (%)	23 (47.9%)

### 3.2 Imaging characteristics

#### 3.2.1 DSA findings

**Figure 2:**
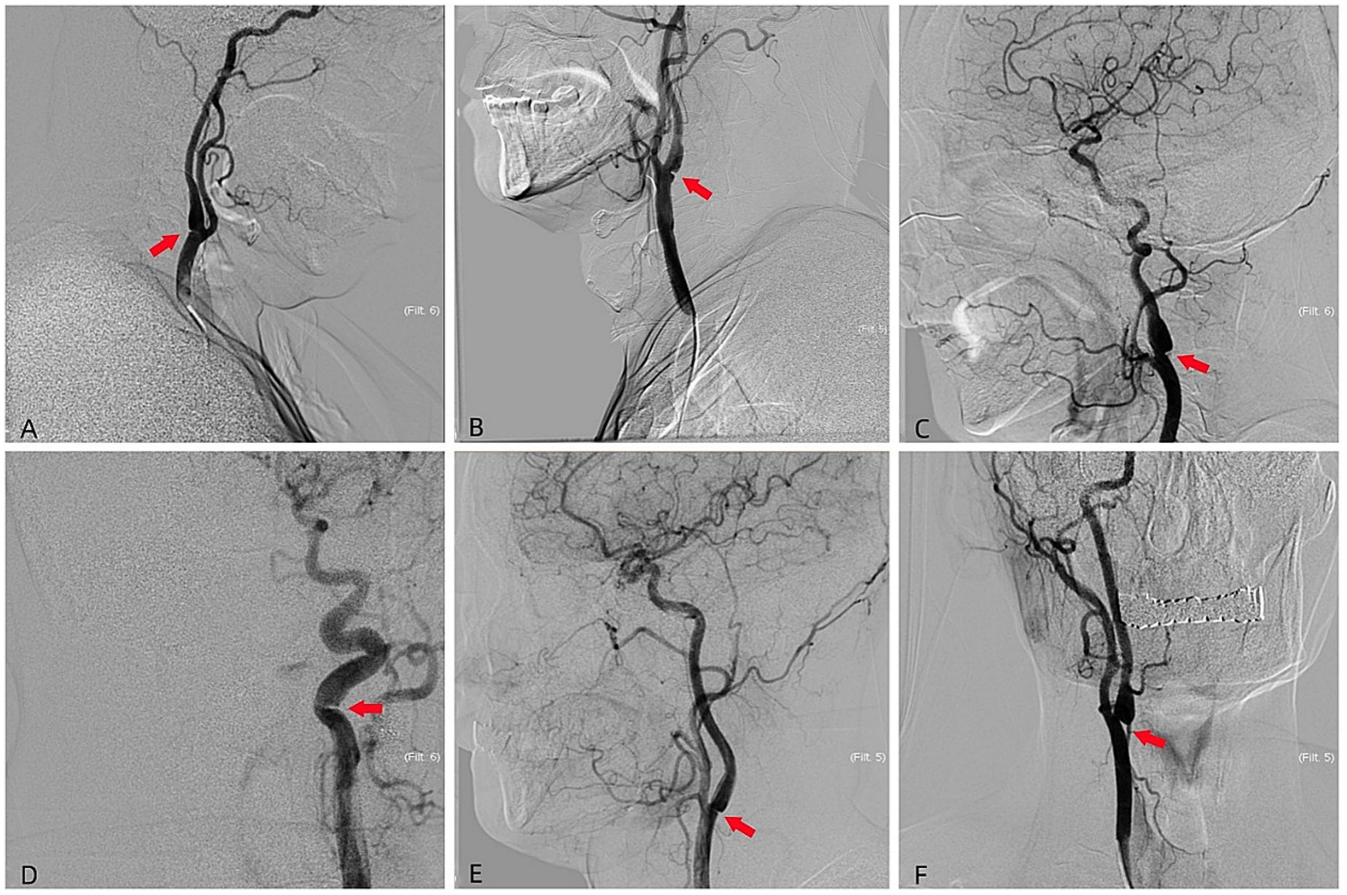
DSA images of some patients. On cerebral angiography, a shelf-like filling defect protruding into the lumen is observed in the proximal internal carotid artery (arrowhead), consistent with the imaging findings of carotid web (CW).

All lesions were located at the carotid bifurcation or the origin of the internal carotid artery on the posterolateral wall. DSA clearly showed a thin, linear or membranous filling defect originating from the vessel wall, with the tip pointing into the lumen, presenting a “ribbon-like” or “fan-shaped” appearance. Among these, 9 cases (18.8%) were combined with mild ipsilateral carotid artery stenosis (<50%). As shown in Figure 2. Of the 48 patients, 10 (20.8%) required adjudication at Step 2 or higher for at least one imaging variable; 3 (6.2%) required third-reviewer arbitration (Step 3 or 4). No case was flagged as “non-resolvable” at Step 5. Inter-observer agreement between the two primary reviewers was assessed by the pre-specified 5-step adjudication algorithm; the proportion of cases requiring adjudication at each step is reported above. Formal Cohen’s *κ* coefficients could not be calculated in this retrospective dataset because the original per-variable reviewer ratings were not stored as separate fields; this will be implemented prospectively in a planned multicenter study.

#### 3.2.2 Subweb thrombus

22 cases (45.8%) met the pre-specified multimodal criteria for subweb thrombus (concordant Doppler + DSA in 21 cases; Doppler + DSA + CEUS in 1 case). Of the remaining 26 cases, 24 had no thrombus on either modality and 2 were classified as indeterminate on Doppler (low-amplitude echoes without a corresponding DSA filling defect) and were therefore not counted as subweb thrombus. Of the 48 patients, 10 (20.8%) required Step 2 or higher adjudication for subweb thrombus; 3 (6.2%) required third-reviewer arbitration; no case was non-resolvable. Inter-observer agreement between the two vascular sonographers was assessed using the same adjudication algorithm; quantitative Cohen’s *κ* coefficients are not reported in this retrospective study but are planned for the prospective phase (see Study Limitations).

#### 3.2.3 Laterality distribution

Left side: 24 cases (50%), Right side: 21 cases (43.8%), Bilateral: 3 cases (6.2%).

#### 3.2.4 Carotid Doppler ultrasound findings

Suggested the presence of CaW in 42 cases (87.5%), no web seen in 6 cases (12.5%).

### 3.3 Treatment

According to the pre-specified institutional MDT protocol described in the Methods section (sub-section 2.3), the 48 patients were allocated as follows: 34 (70.8%) to the medication-only group, 6 (12.5%) to the intervention/surgery group, and 8 (16.7%) to the untreated group. The distribution of indications and outcomes is summarized in Table 3.

**Table 3:** Treatment allocation stratified by indication and outcome.

Group	*n* (%)	Symptomatic CaW (*n*)	Subweb thrombus (*n*)	Recurrent stroke before treatment (*n*)	New stroke during follow-up (*n*)
Medical therapy alone	34 (70.8)	3	16	0	1
Carotid artery stenting	4 (8.3)	4	4	0	0
Carotid endarterectomy	1 (2.1)	1	1	0	0
Emergency thrombectomy	1 (2.1)	1	1	0	0
Untreated (surveillance)	8 (16.7)	0	0	0	0
Total	48 (100)	9	22	0	1

Symptomatic CaW was defined as CaW ipsilateral to a recent ischemic stroke or TIA. Recurrent stroke was defined as a new ipsilateral ischemic event occurring ≥24 h after the index event and after at least 4 weeks of standard antithrombotic therapy. The distribution of symptomatic patients and subweb thrombus across treatment groups reflects the pre-specified MDT criteria (sub-section 2.3): all six patients in the intervention/surgery group met ≥1 criterion for active intervention, and the surveillance group by definition comprised asymptomatic patients without subweb thrombus. All six intervention patients had documented subweb thrombus as the indication for active intervention (subweb thrombus was present in 4 CAS, 1 CEA, and 1 emergency thrombectomy patient; the thrombectomy case additionally presented with acute large-vessel occlusion). The 1 recurrent stroke during follow-up occurred in the medication-only group, consistent with the values reported in the Results section.

*Medication-only group (n = 34, 70.8%)*: All patients received antithrombotic therapy—single or dual antiplatelet therapy (aspirin and/or clopidogrel) or anticoagulant therapy—according to MDT decision. Specifically, aspirin alone was used in 16 cases, aspirin combined with clopidogrel in 13 cases, clopidogrel alone in 4 cases, and anticoagulation alone in 1 case. The indication for medical therapy alone was asymptomatic CaW without subweb thrombus in 15 patients, symptomatic CaW without recurrence in 3 patients, and patient/procedural contraindication to intervention (including high surgical/procedural risk in patients with subweb thrombus who did not meet other intervention criteria) in 16 patients.

*Intervention/surgery group (n = 6, 12.5%)*: All 6 patients met at least one pre-specified criterion for active intervention, namely: recurrent ischemic stroke despite antithrombotic therapy in 0 patients, documented subweb thrombus in 6 patients (with the thrombectomy case additionally meeting the acute large-vessel occlusion criterion; coexisting significant ipsilateral stenosis ≥50% was present in 0 patients). Procedures included carotid artery stenting (4 cases), carotid endarterectomy (1 case), and emergency mechanical thrombectomy (1 case).

*Untreated group (n = 8, 16.7%)*: All 8 patients had asymptomatic CaW without subweb thrombus, and none received antithrombotic therapy; they were managed with clinical and ultrasound surveillance only.

### 3.4 Follow-up results

All 48 patients were enrolled in the standardized follow-up program described in Methods sub-section 2.5. Clinical follow-up was completed in 48/48 patients (100%) at 3 months, in 47/48 (97.9%) at 6 months, and in 46/48 (95.8%) at 12 months; the reasons for the missed visits were documented (predominantly relocation or temporary inability to attend), and no patient was permanently lost to follow-up in the sense of complete withdrawal from all contact with the study team; the two patients who did not complete the in-person 12 month visit remained reachable by telephone and consented to continued passive follow-up. Carotid color Doppler ultrasound was performed in 48/48 (100%) at 6 months and in 46/48 (95.8%) at 12 months; brain MRI was performed in 9 patients (18.8%) in whom a recurrent neurological event was suspected. No cases of recurrent ipsilateral ischemic stroke or TIA occurred in the intervention/surgery group (0/6). One patient in the medication-only group (treated with aspirin plus clopidogrel) experienced recurrent ipsilateral cerebral infarction at month 5, meeting the predefined AHA/ASA 2013 criteria and confirmed by brain MRI with DWI. Among the 8 untreated patients, 1 (12.5%) was found to have new subweb thrombus on carotid Doppler ultrasound at the 6-month visit, without clinical symptoms.

## 4 Discussion

Through the analysis of clinical data from 48 patients definitively diagnosed with CaW by DSA, this study reveals some important characteristics of this condition. The mean age of CaW patients in this study was 60.81 years, with males (54%) slightly outnumbering females. This is similar to findings in relevant Chinese studies. For instance, Zhou et al. (5), in a clinical analysis of 32 CaW cases, found a mean age of (61.3 ± 11.5) years and 18 males (56.25%), which is also consistent with the results of Wang et al. (7). This differs from reports in foreign literature, where most studies indicate that CaW is more common in women aged 45–50, with women accounting for 56.5–76% of patients (5, 7, 35). The prevalence of CaW in the general population is likely less than 1% (9). In a systematic review conducted by Mac Grory et al., the prevalence of ipsilateral CaW in patients under 60 years with cryptogenic stroke was 13% (13). The differences in age and gender distribution of CaW between domestic and international studies may be related to study design, sample size, and ethnicity. The results of this study suggest that CaW does not only occur in young populations; it also needs attention in the etiological screening of cryptogenic stroke in middle-aged and elderly patients. For patients of any age, especially young patients with ischemic stroke lacking traditional risk factors, CaW should be included in the differential diagnosis after excluding other common causes such as cardioembolism and large artery atherosclerosis.

Ischemic stroke patients associated with CaW typically have fewer traditional cardiovascular and cerebrovascular risk factors. A 2018 systematic review showed that 57% of symptomatic CaW patients had no identified cardiovascular or cerebrovascular risk factors (6). In the study by Multon et al. (12), only 4 out of 14 symptomatic patients (36.4%) had at least one atherosclerotic risk factor. Meanwhile, the Brinster series showed that 62% of patients had hypertension, 52% had hyperlipidemia, 40% had a smoking history, only 12% had diabetes mellitus, and only 28% had multiple atherosclerotic risk factors simultaneously (8). The results of this study also show that among the 48 patients, 13 (27.1%) smoked, 12 (25%) consumed alcohol, 17 (35.4%) had hypertension, 18 (37.5%) had hyperlipidemia, 7 (14.6%) had diabetes mellitus, 2 (4.2%) had hyperhomocysteinemia, and 4 (8.3%) had hyperuricemia. The proportions of atherosclerotic factors were generally not high, similar to foreign study results. Nevertheless, some studies have confirmed specific associations between CaW and atherosclerosis or fibromuscular dysplasia (17, 36).

The main reasons for CaW patients seeking medical attention can be divided into three categories: (1) Ischemic stroke or transient ischemic attack, which is the primary and most serious reason (37); (2) Asymptomatic patients discovered incidentally during imaging examinations for other reasons (6); (3) A few cases with atypical neurological symptoms such as dizziness and headache. In this study, the main reasons for CaW patients seeking medical attention were: discovery during carotid ultrasound performed for physical examination or other reasons; followed by discovery during investigation of dizziness and headache; the proportion of patients presenting primarily with ischemic events was 18.8%. Current understanding of asymptomatic CaW remains insufficient.

### 4.1 Asymptomatic carotid web: natural history, risk stratification, and management

In our cohort, 23 of 48 patients (47.9%) were diagnosed incidentally, consistent with the 30–50% reported in previous series (6, 23, 37). In the 8 untreated asymptomatic patients, one developed a new subweb thrombus during follow-up without clinical symptoms, suggesting that surveillance alone may be insufficient in some patients. Risk stratification should be based on imaging biomarkers: subweb thrombus on Doppler or DSA (6, 32), unfavorable web morphology on CFD (32), bilaterality (6), and prior contralateral symptomatic CaW (27, 38). We propose a pragmatic three-tier framework (Table 4): (i) low risk—surveillance every 6 months in year 1, then annually; (ii) intermediate risk—surveillance every 6 months plus low-dose antiplatelet; (iii) high risk—MDT discussion for prophylactic intervention. This framework is hypothesis-generating and requires prospective validation (8, 27, 30, 37).

**Table 4:** Proposed risk-stratification framework for asymptomatic carotid web.

Risk category	Imaging biomarkers	Suggested management (hypothesis-generating)
Low risk	No subweb thrombus, small web, unilateral, no contralateral symptomatic CaW	Surveillance (carotid Doppler every 6 months in year 1, annually thereafter); antiplatelet therapy at clinician discretion
Intermediate risk	Any one of: subweb thrombus (stable), web enlargement, bilaterality, prior contralateral symptomatic CaW	Surveillance every 6 months + low-dose antiplatelet therapy
High risk	Mobile or recurrent subweb thrombus, progressive web enlargement, new ischemic symptoms during surveillance	MDT discussion regarding prophylactic active intervention (CAS, CEA, or surgical web resection) after shared decision-making

CAS, carotid artery stenting; CEA, carotid endarterectomy; CaW, carotid web; MDT, multidisciplinary team. This framework is hypothesis-generating and has not been prospectively validated.

The incidence of unilateral CaW is significantly higher than bilateral CaW. There is no significant difference in incidence between the left and right sides, and a small number of patients can have bilateral CaW simultaneously (6, 14). Approximately 25% of symptomatic CaW patients also have an asymptomatic CaW on the contralateral side (39, 40). The results of this study show left side in 24 cases (50%), right side in 21 cases (43.8%), and bilateral in 3 cases (6.2%), consistent with foreign study results.

There is no unified standard regarding the optimal treatment regimen for CaW. Due to the lack of authoritative recommendations and discrepancies in recent guidelines, the clinical management of CaW tends to be complex (11, 12). Possible treatment options include regular observation and follow-up, medication (antiplatelet therapy and anticoagulant therapy), and surgical treatment (surgical web resection and carotid artery stenting) (27, 28). For symptomatic CaW patients (i.e., those who have experienced ipsilateral ischemic stroke), most patients receive antiplatelet therapy. However, some scholars advocate anticoagulant therapy as a superior option because CaW leads to local carotid blood flow stasis and thrombus formation (32). According to the American Heart Association guidelines, in the absence of randomized study data in the field of CaW, single antiplatelet therapy is a reasonable treatment strategy (if the patient has high-risk TIA or minor stroke, dual antiplatelet therapy for up to 3 weeks followed by single antiplatelet therapy can also be used) (41). Furthermore, for patients with stroke recurrence, treatment modalities such as carotid artery stenting and surgical web resection have also been reported. Joux et al. (42) reported 7 patients treated with surgical web resection (all with a history of ischemic stroke). After a follow-up of (25.3 ± 19.5) months, none had recurrent ischemic stroke, whereas the recurrence rate in the control antiplatelet therapy group was as high as 30%, suggesting that surgical web resection for secondary prevention of ischemic stroke caused by CaW is significantly superior to antiplatelet therapy alone. Carotid artery stenting, as another minimally invasive treatment, has also had its therapeutic effect confirmed by some studies and case reports (29), has also had its therapeutic effect confirmed by some studies and case reports (35). In this study, 4 patients underwent carotid artery stenting, 1 underwent carotid endarterectomy, 1 underwent emergency endovascular thrombectomy, and 34 received medication alone.

It should be emphasized that, in the present study, treatment allocation was not randomized but followed a pre-specified institutional multidisciplinary team (MDT) protocol (see Methods sub-section 2.3). All six patients in the intervention/surgery group met at least one of the predefined criteria for active intervention, whereas none of the 34 patients in the medication-only group fulfilled these criteria. Accordingly, the comparison of outcomes between the intervention/surgery group and the medication-only group should be interpreted in the context of indication-driven selection; residual confounding by indication cannot be fully excluded. Prospective randomized studies are warranted to definitively establish the comparative effectiveness of active intervention versus medical therapy alone in symptomatic CaW patients (22, 30).

In our cohort, no patient in the active intervention group (0/6) experienced a recurrent ipsilateral ischemic event during 3–12 months of follow-up, whereas one patient in the medical-therapy-alone group (1/34, 2.9%) experienced recurrent ipsilateral cerebral infarction. At face value, this difference is consistent with the hypothesis that active endovascular intervention may be associated with a lower risk of stroke recurrence than medical therapy alone in symptomatic CaW patients or in those with subweb thrombus. However, this observation does not establish a causal relationship, for the following reasons: (i) the comparison is unadjusted and confounded by indication, since intervention was offered only to patients who fulfilled pre-specified criteria for active treatment (sub-section 2.3); (ii) the sample size is too small (*n* = 6 vs. *n* = 34) to permit any meaningful statistical test of between-group differences; (iii) follow-up is short (3–12 months), precluding assessment of durable treatment effects; and (iv) the comparison is single-center and non-randomized. As a consequence, the data reported here are observational and hypothesis-generating, and no causal inference regarding treatment efficacy can be supported. Properly powered, prospective randomized controlled trials are required to determine whether active endovascular intervention or surgical web resection reduces the risk of stroke recurrence compared with medical therapy alone in this population (22, 30).

For asymptomatic incidental discoveries, due to the lack of reports on the natural history of CaW cases, there is no consensus on the management of asymptomatic patients. The optimal management strategy still requires larger sample prospective studies for confirmation. For these patients, a tiered surveillance strategy is recommended, consisting of clinical evaluation and carotid color Doppler ultrasound (with MRA or CTA as confirmatory imaging when needed) every 6 months during the first year and annually thereafter, combined with low-dose antiplatelet therapy in patients with intermediate-risk imaging biomarkers (subweb thrombus, larger web, bilaterality, or prior contralateral symptomatic CaW). Prophylactic active intervention may be considered only in high-risk asymptomatic lesions (e.g., mobile subweb thrombus, progressive web enlargement, or new ischemic symptoms during surveillance) after multidisciplinary team discussion and shared decision-making with the patient, weighing individual procedural risk against the unproven but biologically plausible benefit of stroke prevention. There is no consensus on the treatment plan and management strategy for stroke caused by CaW, and more evidence-based medical evidence is needed.

### 4.2 Why causal inference cannot be drawn from the present data

Applying the Bradford-Hill criteria (43) to the present data: temporal sequence is plausible and biological plausibility is supported by hemodynamic studies of CaW (32). However, several criteria remain unmet: (i) strength of association is uncertain given the small intervention group; (ii) consistency cannot be assessed because published evidence is methodologically heterogeneous; (iii) specificity is undermined by multifactorial stroke etiology; (iv) biological gradient cannot be evaluated; and (v) experimental evidence (randomized intervention) is absent (43, 44). Therefore, the present findings should not be interpreted as evidence of a causal treatment effect. Only prospective randomized controlled trials can satisfy the Bradford-Hill criteria for causation.

### 4.3 Study limitations

This study is a single-center retrospective study with a limited sample size. Treatment grouping was non-randomized, potentially introducing selection bias. Follow-up duration varied, and long-term efficacy requires further observation. In addition, although treatment allocation followed a pre-specified institutional MDT protocol, the non-randomized design inevitably introduces indication bias (45), and the relatively short follow-up (3–12 months) limits inference regarding long-term therapeutic efficacy. Consequently, no causal inference can be drawn regarding the efficacy of active intervention versus medical therapy alone, and the apparent difference in recurrence rates should not be interpreted as a treatment effect. Finally, formal inter-observer agreement (Cohen’s *κ*) for each imaging variable could not be calculated in this retrospective study because per-variable reviewer ratings were not retained as separate data fields; the planned prospective phase will capture these ratings systematically and report formal κ statistics. The 5-step adjudication algorithm described in sub-section 2.7 nonetheless provides a transparent, auditable record of how each discrepant case was resolved.

## 5 Conclusion

Carotid web is an important causative factor for cryptogenic stroke across all age groups, commonly found at the origin of the internal carotid artery, and is also not uncommon in middle-aged and elderly patients.

In our cohort, the apparent prevalence of subweb thrombus (45.8%) was high; however, this estimate is subject to potential operator-dependent overdiagnosis on Doppler ultrasound and should be interpreted cautiously. Subweb thrombus appears to be a plausible mechanism linking CaW to embolic events and warrants careful assessment in every patient, ideally with multimodal imaging confirmation.

DSA is the “gold standard” for diagnosing and evaluating carotid web, capable of clearly displaying its characteristic morphology and complications. Doppler ultrasound can serve as a preliminary screening tool for carotid web.

Treatment strategies for CaW should be individualized. Given the non-randomized design, small intervention group (*n* = 6), and short follow-up (3–12 months), our findings can only suggest—not confirm—that endovascular intervention or surgical web resection may be associated with a lower risk of stroke recurrence in symptomatic patients or those with subweb thrombus. These observations are hypothesis-generating and require validation in adequately powered prospective randomized controlled trials. For asymptomatic patients, a tiered surveillance strategy is recommended (Doppler every 6 months in year 1, annually thereafter), with low-dose antiplatelet therapy in intermediate-risk patients and MDT discussion for prophylactic intervention in high-risk lesions (Table 4).

## Data Availability

The datasets presented in this article are not readily available because of ethical and privacy restrictions. Requests to access the datasets should be directed to dingzhejing2011@qq.com.
